# Addressing emerging public health threats: the Noncommunicable Disease Capacity Assessment and Planning (N-CAP) Process

**DOI:** 10.3389/fpubh.2024.1384957

**Published:** 2024-06-05

**Authors:** Randa K. Saad, Ruba Alsouri, Meredith H. Kruse, Lara Kufoof, Sophie Lobanov-Rostovsky, Patricia Richter, Yousef Khader

**Affiliations:** ^1^Research and Policy, Center of Excellence for Applied Epidemiology, Global Health Development | Eastern Mediterranean Public Health Network, Amman, Jordan; ^2^Workforce Capacity, Center of Excellence for Applied Epidemiology, Global Health Development | Eastern Mediterranean Public Health Network, Amman, Jordan; ^3^Global Public Health Systems, Division of Global Health Protection, United States Centers for Disease Control and Prevention, Atlanta, GA, United States; ^4^Alexton Inc., Springfield, VA, United States; ^5^Project Management Office, Global Health Development | Eastern Mediterranean Public Health Network, Amman, Jordan; ^6^Department of Community Medicine, Public Health, and Family Medicine, Faculty of Medicine, Jordan University of Science & Technology, Irbid, Jordan

**Keywords:** noncommunicable diseases, public health, N-CAP Process, multisectoral, assessment and planning

## Abstract

**Background:**

The global epidemic of noncommunicable diseases (NCDs) is increasing. Current assessments that monitor capacity to address NCDs are often externally led and do not facilitate country planning. The Noncommunicable Disease Capacity Assessment and Planning (N-CAP) Process assists ministries of health and other governmental and non-governmental stakeholders to assess, prioritize, and plan how to address NCDs and other public health threats. This paper describes the development of this tool.

**Materials and methods:**

Driven by ministries of health, the N-CAP Process engages new and existing stakeholders in three activities: Stakeholder Mapping; Strengths, Weaknesses, Opportunities, and Threats Workshop; and N-CAP Workshop that uses Discussion Guides to lead in-depth assessment and planning. Standard Operating Procedures, a library of Discussion Guides based on common NCD themes, and an open-access e-learning course are available.

**Results:**

The N-CAP Process outcome is a prioritized plan of how to improve the country’s public health functions. Adaptations to the tool were made after piloting in Jordan and Iraq.

**Conclusion:**

The N-CAP Process helps countries engage various stakeholders to identify gaps and create collaborative, country-specific strategies to effectively respond to NCDs, a leading public health threat. The pilots sparked interest from other countries and underscored its potential for broader implementation to combat the rising global burden of NCDs.

## 1 Introduction

Noncommunicable diseases (NCDs), including cancer, chronic respiratory illnesses, cardiovascular diseases, and diabetes, cause 74% of global mortality, or more than 7 out of every 10 deaths (1). The importance of addressing NCDs was highlighted during the COVID-19 pandemic, as the world witnessed disproportionate rates of hospitalization and death among patients diagnosed with coronavirus disease and living with pre-existing NCDs (2, 3). Certain prevalent NCDs and their risk factors, such as diabetes, cardiovascular disease, and hypertension, were associated with an increased risk of SARS-CoV-2 infection and progression to more severe coronavirus disease with poorer outcomes (4), underscoring the threat that NCDs pose to global health security (5). Public health systems were ill-prepared to respond to an emergent public health threat and maintain continuity of care for chronic diseases, especially in low- and middle-income countries that were less likely than upper-middle and high-income countries to include NCDs as essential services in COVID-19 emergency response plans (6).

In 2015, world leaders pledged to achieve target 3.4 of the United Nations’ Sustainable Development Goals (SDGs) to reduce one-third of premature mortality from NCDs by 2030 (7). Although the rate of premature mortality from NCDs is declining globally, the pace is too slow to reach the SDG target (8). Based on 2010–2016 trends, only 15 countries are on track to reach this target for men and 17 for women (8). The World Health Organization’s (WHO’s) NCD Progress Monitor and NCD Country Capacity Survey are periodic assessments that evaluate progress toward achieving NCD-related indicators and capacity to respond to NCDs (9, 10). These are invaluable tools led by the WHO and based on global data or questionnaire responses from country focal points. However, lacking from these assessments is the country’s internal evaluation and planning on how to function more effectively to make greater progress. New tools and processes that complement existing assessments and surveys are needed to help countries internally assess their capacities and develop collaborative, context-specific plans to better tackle the burden of NCDs and other public health threats.

The Office of Global Noncommunicable Diseases (Global Health System Strengthening Team as of October 2023) within the United States Centers for Disease Control and Prevention (CDC) partnered with the CDC’s National Public Health Institute Program, the International Association of National Public Health Institutes (IANPHI), and Global Health Development|Eastern Mediterranean Public Health Network (GHD|EMPHNET) to develop the Noncommunicable Disease Capacity Assessment and Planning (N-CAP) Process (11). The N-CAP Process is a tool that assists ministries of health and other governmental and non-governmental stakeholders to assess, prioritize, and plan how to strengthen public health functions so that countries can more effectively respond to the NCD epidemic.

This paper outlines the development of the N-CAP Process and insights gained from pilot implementation in the Eastern Mediterranean Region, which helped revise and validate the Process. We describe how a collaborative, multisectoral approach to address NCDs was conceptualized, implemented, and refined based on real-world applications. We provide health policymakers, program managers, and public health professionals with the description of a validated tool that can be adapted to their specific local needs and context, offering an accessible resource that can enhance the effectiveness of the NCD response globally.

## 2 Materials

Numerous materials were developed to support the implementation of the N-CAP Process (11). This included Standard Operating Procedures (SOPs), Discussion Guides (DGs), and N-CAP Workshop Forms. The development of this material was to ensure consistency in application and provide the flexibility needed to tailor the tool to specific local contexts, reflecting the global applicability of the N-CAP Process.

### 2.1 Standard operating procedures

To standardize implementation in different countries and regions, while allowing for necessary adaptations, a comprehensive set of SOPs was created. The SOPs comprise modules for each activity within the N-CAP Process (Supplementary material), including an Introduction and Preparation module which provides an overview of the entire N-CAP Process and preparatory steps. Each module defines roles and responsibilities and describes the procedures and materials used, ensuring that a clear framework is followed. Appendices within these SOPs provide additional resources, such as forms and report templates, which are crucial for documenting and guiding the activities effectively.

### 2.2 Discussion guides

DGs were created to facilitate in-depth discussions during the N-CAP Workshop, described below. The guides are tailored to address common themes countries face when strengthening NCD efforts. Currently, seven DGs (Table 1) are available, including a “General NCD Efforts” DG that can be used when no other DG aligns with the critical NCD areas identified by a country.

**Table 1:** N-CAP Process discussion guides.

Discussion guide	Purpose
Coalitions	To develop a new or strengthen an existing NCD coalition that efficiently uses resources to achieve greater impact
Evidence-informed action	To use scientific data and other information to develop programmatic and/or policy recommendations and guide implementation
General NCD efforts	To assess and improve overall efforts to identify and address the country’s NCD priorities
Health communications	To assess and improve health communications efforts for increased impact
Multisectoral action	To assess and improve collaborative actions among multiple sectors for the prevention and control of NCDs
Strategic data collection and analysis	To ensure data collection and analysis are strategic, and the data and other information are integrated and shared in ways that inform public health policy and programs
Surveillance	To ensure strong systems for collecting and analyzing data using surveillance, surveys, and other methods

NCD, Noncommunicable diseases; NGOs, Non-governmental organizations.

DGs describe the level of development or progress that a country is at with respect to an identified critical NCD area. They use the conceptual framework of a maturity model that defines increasing levels of “maturity” or progress. Tables 2, 3 describe the four levels of progress (Beginning, Progressing, Advanced, and Leading Edge) for six broad public health domains (Strategic Direction, Systems, Resources, Quality, Engagement, and Impact). The range of issues covered by the domains helps ensure a systematic approach, enhancing the depth and utility of the discussions.

**Table 2:** N-CAP Process discussion guide levels of progress.

Level of progress	Definition
Beginning	Countries are at a beginning level of progress in the relevant domain
Progressing	Countries are developing processes and systems to strengthen their progress
Advanced	Countries have well-established processes and systems in place to support their efforts
Leading edge	Countries have robust systems in place and are employing innovative solutions

**Table 3:** N-CAP Process discussion guide domains.

Domain	Considerations
Strategic direction	Are the priorities clear and strategic?
Systems	Are the necessary tools, processes, etc. in place to accomplish the work?
Resources	Are human and material resources adequate?
Quality	Is quality measured and are standards met?
Engagement	Are the key stakeholders engaged?
Impact	Are the efforts contributing to improve population health related to NCDs?

### 2.3 N-CAP Workshop Forms

Various forms were developed to record and organize discussions during the N-CAP Workshop. These include the Assessment Form, which documents the levels of progress and rationale of the participants, the Prioritization and Planning Form that reflects the next steps participants agree to help progress the country, and the optional External Evaluator Form that can be used to evaluate how the Workshop was led. Additional information about how to effectively use these forms is described in detail in the SOPs (11).

## 3 Methods

**Figure 1:**
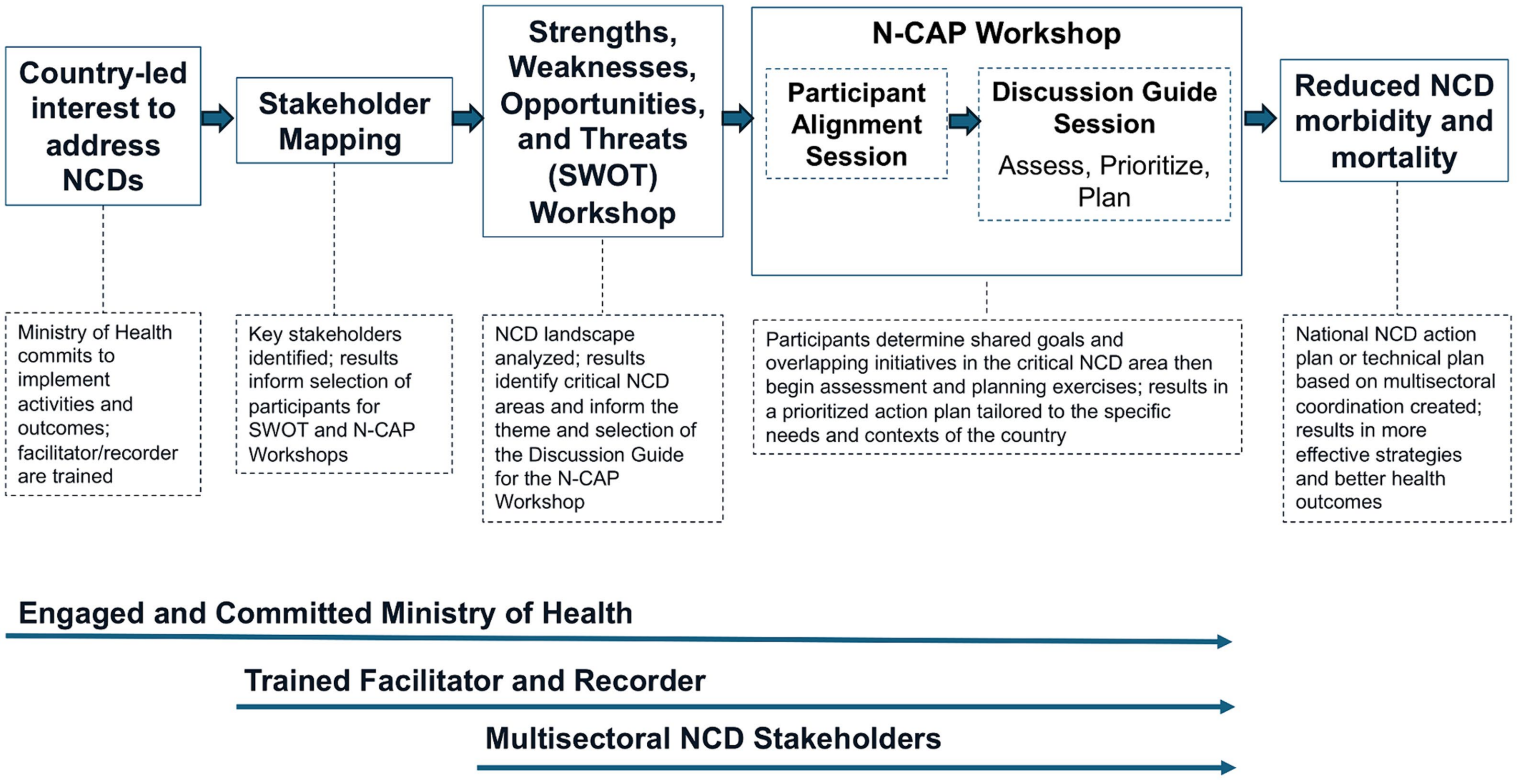
Conceptual framework of the Noncommunicable Disease Assessment and Planning (N-CAP) Process.

The N-CAP Process was created as an assessment and planning resource that supports national-level efforts to address NCDs. Figure 1 depicts the conceptual framework for the N-CAP Process. This methodology paper outlines the systematic approach used in the development of the N-CAP Process, detailing the design, piloting, and refining stages that contributed to the final Process.

### 3.1 Development and design

The N-CAP Process was conceived through a collaborative effort with the CDC, EMPHNET, and IANPHI. It is based on the Staged Development Tool (SDT) which was developed jointly by the CDC and IANPHI to help national public health institutes (NPHIs) assess their current capacity and develop a plan to reach a higher level of functioning (12). The SDT is typically conducted as part of the creation or expansion of an NPHI or as part of an effort by the NPHI to improve functioning in specific areas (12).

The N-CAP Process was designed to meet the specific demands of NCD prevention and control and other public health threats and was developed to be led by the Ministry of Health with engagement from a range of stakeholders from multiple organizations. It includes three facilitator-led sequential activities: (1) Stakeholder Mapping; (2) Strengths, Weaknesses, Opportunities, and Threats (SWOT) Workshop; and (3) N-CAP Workshop (11).

Each activity concludes with a summary report, which informs the next activity in the N-CAP Process and serves as a resource for the Ministry of Health. The final N-CAP Process Report provides an overall summary of all activities, outputs, and outcomes, and can assist the country and stakeholders in developing an integrated, multisectoral national NCD policy, strategy, and/or action plan or a country-specific technical plan to tackle particular aspects of NCD prevention and control. The N-CAP Process can be repeated by a country to assess progress made since the initial assessment or to focus on an additional NCD critical area.

### 3.2 Defining each activity

The N-CAP Process activities start with Stakeholder Mapping. Facilitators consider key technical and policy stakeholders based on their level of interest in contributing to NCD efforts, and their level of influence or responsibility for finances, policies, or other resources needed to address NCDs. The Stakeholder Mapping Report documents the results of the mapping activity, includes recommendations of participants for the SWOT Workshop, and later informs the selection of participants for the N-CAP Workshop.

The SWOT Workshop is the second activity of the N-CAP Process. Facilitators lead a candid discussion with participants about the NCD landscape within the country. Participants identify strengths that can be leveraged, weaknesses that should be addressed, opportunities that can be capitalized on, and threats that must be considered. The discussions are summarized in the SWOT Workshop Report, which also describes critical NCD areas within the country that inform the theme of the subsequent N-CAP Workshop. Critical areas identified during the SWOT are matched to DGs. During the N-CAP Workshop, one or more DGs can be used, and different individuals may participate in different DG sessions.

The N-CAP Workshop is the last activity in the N-CAP Process. It is a multi-day workshop led by the facilitators. The N-CAP Workshop begins with an Opening session that sets the stage for the discussions. Next is the Participant Alignment session that encourages participants to envision how they can work together more effectively in new or existing relationships. The session facilitates a discussion of the work and goals of each participating organization to identify overlapping and complementary efforts. It prompts participants to consider how their work fits into the country’s overall NCD strategy and response. It also helps participants, who may be meeting for the first time or be unfamiliar with each other’s work, to begin to think as a team with the common goal of reducing the burden of NCDs in their country and addressing related public health threats.

The N-CAP Workshop then continues to the DG Session that includes the Assessment Phase, Transition, Prioritization and Planning Phase, and Summary Wrap-Up (Table 4). A highly structured DG is used during the Assessment Phase to facilitate customized and in-depth discussions. Participants determine the country’s overall level of progress within the critical NCD area, and then assess the country’s current and preferred level of progress in each of the six domains. Participants may not agree on the country’s level of progress across the different domains; however, the content of the discussion is more important than unanimity on the level of progress. During the Prioritization and Planning Phase, participants identify efforts needed to reach an advanced level of progress. The Workshop concludes with a prioritized plan of next steps to move the country forward to a higher level of functioning.

**Table 4:** Phases of the discussion guide session.

Phase	Objective	Approach
Assessment	Assess the current level of progress and the preferred level for the country in each of the six domains of the DG	Participants discuss the country’s overall level of progress related to the critical NCD area/DG theme. This is repeated for each of the six domains with participants providing reasoning/justification for their choice. Participants then discuss their preferred level of progress for each domain and the possible next steps required to reach the preferred level. The discussion is recorded in the Assessment Form.
Transition	Create an organized document that reflects the Assessment Phase discussion and prepares participants for the Prioritization and Planning Phase	Participants are not directly involved. Facilitators and recorders review the Assessment Form and group similar ideas together to create categories or related issues that can be solved by connected next steps. This is recorded in the Prioritization and Planning Form.
Prioritization and planning	Create a prioritized plan of next steps needed to move the country to a more advanced level of progress within the identified critical NCD area	Categories, details, and next steps listed in the Prioritization and Planning Form are reviewed and prioritized by the participants as the most essential to achieving a higher level of functioning. Potential “quick wins,” or efforts that can be easily and generally quickly achieved, are identified. The discussion then progresses to create a more complete next steps plan, including ownership/ assigned responsibility and timelines to achieve the efforts^1^.
Summary wrap up	Summarize the N-CAP Workshop and agree on follow-up	Facilitator summarizes the completed Prioritization and Planning Form. The public health organization discusses plans for follow-up. The Workshop concludes.

DG, Discussion Guide; NCDs, Noncommunicable diseases.

^1^If the country has not yet identified ownership/ assigned responsibility and timelines, the Prioritization and Planning Form can be used to capture a prioritized list of recommendations that can inform the development of a technical plan in the future.

After the N-CAP Process activities conclude, a final N-CAP Process Report is written, as previously described.

### 3.3 Defining roles and responsibilities

Ministries of health are the leading stakeholder and their leadership is vital to the success of the N-CAP Process. The N-CAP Process begins when the interested Ministry of Health and the national or regional public health organization, such as a regional public health network, field epidemiology training program, or NPHI, begin discussions. Ongoing follow-up and coordination are essential to ensure the Ministry of Health is willing and able to commit the necessary time and resources for sustained participation. A readiness criterion was developed (Table 5) to identify and assess the capacity of ministries of health that are ready for effective implementation of the N-CAP Process. Ministries of health are then continuously engaged to encourage ownership of the activities and implementation of outcomes.

**Table 5:** Criteria to determine the ministries of health best prepared to conduct the N-CAP Process.

Component	Criterion
Current NCD efforts	MoH is currently working to improve the ways in which they address NCDs in the country
NCD departments	Departments exist that work on NCDs (e.g., CVD, cancer, health systems, etc.) and regularly collaborate and work with other departments for strategy development and program implementation
Engagement	Key MoH staff recognize the importance of engagement with non-governmental stakeholders and are open to this type of multisectoral collaboration
Capacity and willingness	Key MoH staff have the capacity to play an active leadership and decision-making role in the N-CAP and are willing to commit necessary resources to ensuring the activities’ success

CVD, Cardiovascular diseases; MoH, Ministry of Health; N-CAP Process, Noncommunicable Diseases Capacity Assessment and Planning Process; NCDs, Noncommunicable disease.

In collaboration with the Ministry of Health, public health organizations also play a vital role in facilitating the N-CAP Process. This begins with the joint selection of key personnel, including a facilitator and a recorder, who are tasked with leading the activities of the N-CAP Process. The Ministry of Health appoints a Point of Contact (POC) within its NCD department, or equivalent, with the decision-making authority to actively lead the N-CAP Process alongside the selected facilitators.

### 3.4 NCD stakeholders as participants

The Ministry of Health liaises with other country or regional stakeholders who can contribute their expertise to the discussion, planning, and resources needed to facilitate outcomes of the N-CAP Process. The Ministry of Health works closely with the facilitator to identify appropriate multisectoral organizations to engage in the N-CAP Process activities. The organizations then nominate their representatives to participate. Participants are asked to speak to the organization’s capacity to contribute to the larger effort to address NCDs and have knowledge of how to facilitate action.

Participants have different levels and types of expertise based on the activity. For the SWOT Workshop, NCD-focused governmental and non-governmental organizations who work on general NCDs, specific diseases, risk factors, or behavior change initiatives are invited. These organizations should have both an interest in contributing to efforts to address NCDs and the ability to influence efforts to address NCDs because of their financial, political, or other resources. For the SWOT Workshop, participants do not need to be in a leadership role. An example of the ideal participant might be the Operations Manager, Deputy Executive Director, etc. For the N-CAP Workshop, more technical expertise is required by participants. Organizations with expertise in the DG theme used for the Workshop are invited to attend. These participants have a leadership role with decision-making authority and access to the resources needed to act on the outcomes of the N-CAP Workshop. An example of the ideal participant might be the Director, Chief Executive Officer, etc.

The public health organization either works with the Ministry of Health to identify the facilitators and recorders or can serve in this role itself. It also can be considered a participant in the activities. The roles the public health organization occupies may vary as appropriate to its resources, structure, and country context. In countries in which the public health organization is neither a facilitator/recorder or participant, it can observe the N-CAP Process activities to ensure it is informed of the outcomes and results. This involvement helps the public health organization build contacts with the different stakeholders, including the Ministry of Health. It also follows up with the Ministry of Health and the participants after the N-CAP Process is completed to receive feedback on successes and challenges in implementing the next steps.

### 3.5 Trained facilitators and recorders

All N-CAP Process activities are led and conducted by trained facilitators and recorders. It is beneficial for facilitators to be knowledgeable about disease prevention and control and general public health issues and have experience moderating meetings with participants of various levels of authority. Recorders assist the facilitators by documenting the discussions during the two workshops. The facilitators and recorders do not provide any input or opinion on the topics discussed during workshops to guarantee neutrality. Multiple facilitators and recorders can be trained; however, it is recommended that one or more facilitators and recorders are used, and the same individuals continue throughout the entire N-CAP Process.

Prior to conducting any activity, the facilitators and recorders must complete the N-CAP Process Facilitator and Recorder Training, which is available as a self-guided, open-access e-learning course (13). The e-learning training course provides an overview of procedures and facilitation best practices and uses interactive exercises to prepare the learner to implement each activity of the N-CAP Process.

### 3.6 Pilot implementation

The N-CAP Process was piloted in English and Arabic in two countries in the Eastern Mediterranean Region (EMR), Jordan and Iraq, respectively. The EMR was identified through conversations with the CDC and local partners as a region interested in strengthening their NCD response. The EMR, with an estimated population of 779.7 million people in 2022 (14), has the highest rate of premature mortality due to one of the four main NCDs (24.5%, UI, 16.7–34.0), compared to all other world regions (15). In 2019, the percentage of premature mortality from the four main NCDs was 15.3% in Jordan and 23.5% in Iraq (16).

In this section, we briefly describe how the outcomes from N-CAP Process pilots in Jordan (2021) and Iraq (2022) informed revisions to the tool and activities. Additional details about the implementation of each pilot and outcome are described in a forthcoming manuscript.

In 2021, the CDC and IANPHI virtually trained four representatives from GHD|EMPHNET and the Royal Health Awareness Society (RHAS), a local partner in Jordan, as facilitators (*n* = 2) and recorders (*n* = 2). The N-CAP Process was implemented by GHD|EMPHNET and RHAS in collaboration with the Jordanian Ministry of Health, with participation from the WHO and other NCD stakeholders in Jordan. The N-CAP Workshop focused on the “data-to-action continuum,” and assessed how the country collects and analyzes NCD data and synthesizes information to inform programs and policy. Results were published by GHD|EMPHNET as a policy brief that emphasized the need for a national NCD strategy that addresses data priorities, including a health information system for NCD data collection (17). The Workshop outcomes later informed aspects of the Ministry of Health’s “Roadmap for Strengthening Primary Care in Jordan” and the Ministry of Health’s “NCD Strategy and Action Plan,” which are both currently being developed.

In 2022, GHD|EMPHNET began conversations with the Iraq Ministry of Health as a potential country to lead the second pilot of the revised N-CAP Process. Iraq was identified due to its interest in NCDs and its highly motivated NCD Department who was eager to implement the tool. GHD|EMPHNET identified two local consultants with experience in leading and facilitating high-level meetings. GHD|EMPHNET supported their training as N-CAP Process facilitator and recorder and provided technical assistance, under the guidance of the Iraqi Ministry of Health and in collaboration with CDC and IANPHI. The SWOT Workshop identified the most important priority was strengthening an existing NCD coalition. Therefore, the N-CAP Workshop in Iraq brought the coalition members together to use the Coalition DG to assess and plan how to meet this goal. The plan of next steps addressed internal communication strategies, tackled the issue of finding new sources of funding to implement joint activities, and focused on efforts to update and review legislation related to the promotion of healthy diet and physical activity. Since implementation, the coalition has held regular meetings and begun to evaluate targeted legislation.

## 4 Results

The results described here detail the expected outcomes of the N-CAP Process activities when implemented by a country and Ministry of Health. We then recount the lessons learnt from the pilots in Jordan and Iraq and demonstrate how the final N-CAP Process was refined based on these experiences.

### 4.1 Overview and expected outcomes

The developed N-CAP Process facilitates assessments and strategic planning to enhance the multisectoral response to NCDs. By engaging a wide range of stakeholders and utilizing a structured, modular approach, this Process helps to identify and prioritize actions to strengthen public health functions and combat NCDs more effectively.

The outcome of the N-CAP Process is a completed assessment and prioritized plan of how to improve the country’s public health functions and collaboratively respond to public health threats. Execution of the plan is the responsibility of the government. The Ministry of Health and stakeholders are encouraged to use the findings and next steps identified during the N-CAP Workshop to inform policymaking and/or to establish technical plans to implement the next steps. They can develop a multisectoral, nationally integrated NCD and risk factor policy, strategy, and/or action plan. They can also construct comprehensive technical plans to address a particular component of NCD prevention and control within their country.

### 4.2 Lessons learnt from pilots

The N-CAP pilots in Jordan and Iraq demonstrated its feasibility and effectiveness in enhancing NCD prevention and control efforts. Following the pilots, several modifications were made to the N-CAP Process materials and procedures to better align with local needs and enhance usability.

Piloting the N-CAP Process in Jordan led to revisions to the materials, approach, and activities. First, specific N-CAP Process materials, including the SOPs and additional DGs previously described, were created to standardize, and guide implementation across diverse settings. Next, during the N-CAP Workshop, it became evident that earlier and ongoing engagement with the Ministry of Health is crucial for collaboration and achieving desired outcomes. Their initial hesitance highlighted the importance of involving them right from the planning stages through to the execution of the N-CAP Workshop. This learning informed the conceptualization of how countries and ministries of health should be approached to use the N-CAP Process and is reflected in the content of the SOPs.

The Jordanian pilot also highlighted the need for some procedural changes to the N-CAP Workshop, which was initially based on the workshop structure from the SDT (12). Changes were needed due to differences in the way the SDT and N-CAP Process are used. For instance, the SDT was designed to be used within a single organization (12), while the N-CAP Process brings together participants from a variety of organizations who may not be familiar with each other prior to the activities (11). In Jordan, the participants had existing relationships, but it was necessary to re-introduce all in order to help the participants begin to think collaboratively. To address the additional challenges that having participants from multiple organizations presented, the Participant Alignment session was developed. As described in 3.1, the Participant Alignment session takes place after the Opening of the N-CAP Workshop. It sets the stage to encourage participants to envision how they can work together for greater impact.

The Iraq pilot led to revisions in the recruitment and training of facilitators and recorders. Unlike the pilot in Jordan which was led by GHD|EMPHNET staff, the Iraq pilot was led by local, external facilitators. GHD|EMPHNET recognized the importance of having representatives with existing relationships with the Ministry of Health and local stakeholders to secure “buy-in” and effectively facilitate the activities. Using local facilitators and recorders who were familiar with the unique NCD landscape in the country helped to strategically lead the discussions and anticipate potential areas of disagreement. A recommendation about the importance of locally led facilitation was included in the revised SOPs.

Similarly, the need for accessible training for facilitators and recorders was apparent. Like in Jordan, the Iraqi facilitators and recorders were virtually trained by GHD|EMPHNET and CDC. However, this was recognized by CDC and GHD|EMPHNET as an unsustainable format that would not be easily scalable to a global audience. To reduce barriers to training, the CDC, GHD|EMPHNET, IANPHI, and the Training Programs in Epidemiology and Public Health Interventions Network (TEPHINET) adapted the prior virtual trainings and developed the N-CAP Process Facilitator and Recorder Training Course e-learning (13).

The results from the pilot implementations informed critical revisions to the N-CAP Process, which is now publicly available for use by any Ministry of Health seeking to improve public health functions and better address NCDs.

## 5 Discussion

The N-CAP Process pilots garnered interest from other countries, including Pakistan which began implementation in 2023, demonstrating the usability of the tool in any country around the world. For example, Jordan and Iraq had different socioeconomic structures and burdens of NCDs and public health priorities, yet both were able to successfully adapt the N-CAP Process to meet their specific needs.

The pilot experiences offer valuable lessons for other countries grappling with the growing burden of NCDs. These lessons, garnered from real-world applications, highlight strategies that can be adapted to diverse health systems across the globe. The pilots demonstrated the N-CAP Process’ flexibility that allowed it to be effectively customized to meet local needs. In Jordan, adaptations were made to address specific data gaps in NCD surveillance, while in Iraq, the focus was on strengthening existing NCD coalitions. This adaptability underscores the importance for other countries to tailor frameworks to their unique socio-political and health landscapes, enhancing the relevance and impact of such interventions. The scalability of the N-CAP Process makes it an ideal tool for countries at different stages of public health development. Whether a country is looking to initiate, expand, or refine its NCD response, the N-CAP Process provides an accessible model that can be adjusted according to the scope and scale of local needs. It can also be used repeatedly to assess progress made over time.

Critical to the N-CAP Process is the engagement of multisectoral stakeholders and the establishment of new or strengthening of existing partnerships for greater impact. There are often key stakeholders, such as multilateral organizations, government ministries, non-profit organizations, academics, and others who play a role in addressing NCDs, but may work in siloes with limited collaboration. As a result, many NCD efforts do not reach their potential capacity for impact. The N-CAP Process facilitates strategic coordination and collaboration to more effectively respond to public health threats. A key takeaway from the pilots was the critical importance of involving a broad array of stakeholders early in the planning process. This engagement ranged from government ministries to non-governmental organizations and community representatives, ensuring that the strategies developed were inclusive and comprehensive.

Unlike previous NCD-related workshops and assessment tools, the N-CAP Process is a country-owned assessment and planning exercise. Because of this, it has several strengths. It is driven by the Ministry of Health leadership and builds on the expertise, experiences, and perspectives of stakeholders knowledgeable and engaged in the country’s approach to addressing NCDs. It does not rely on outside experts and depends on the active engagement and partnership of stakeholders to support the next steps that will help move the country forward to a higher level of functioning. The N-CAP Process allows the country to identify and prioritize the critical public health areas based on the discussion and input of multiple stakeholders during the SWOT Workshop. Accordingly, the priorities are context-specific and not based on external or funder preferences. The N-CAP Workshop encourages new and existing stakeholders to collaboratively assess the progress that their country has made in addressing NCDs by evaluating several aspects of their response against an impartial set of benchmarks. The assessments, discussions, and plans that are developed are determined by the participants, not external entities or funders. Additionally, securing “buy-in” and active participation of the Ministry of Health encourages implementation and more sustainable outcomes.

The N-CAP Process has a number of limitations that should be accounted for by countries considering implementation. The N-CAP Process requires strong facilitators to manage discussions with multiple stakeholders and help them identify underlying reasons behind barriers, challenges, or bottlenecks that prevent the country from effectively responding to the NCD epidemic. Facilitators remain neutral to the discussion, but must be well aware of public health issues. Even though plans may be comprehensive, it is the responsibility of participants to implement them, otherwise the plans stay on paper. Monitoring implementation and accountability relies on strong Ministry of Health governance, which in certain countries is affected by high leadership and staff turnover.

While countries must account for these limitations, a well-implemented N-CAP Process led by strong facilitators and supported by the Ministry of Health ensures the involvement of influential and motivated stakeholders and results in an actionable plan. These plans include roles and responsibilities of stakeholders that help leadership to monitor progress, ensure accountability, and increase the likelihood that plans are implemented. The DGs and N-CAP Workshop structure also enable the diverse stakeholders to consider potentially new and innovative ways to collaborate and work together more effectively.

## 6 Conclusion

The burden of NCDs is increasing within populations throughout the world. It is essential that countries strategically plan and prepare to address public health threats and limit preventable deaths. To be effective, plans should be country-specific, tailored to country priorities, led by ministries of health, and engage the expertise and influence of key NCD stakeholders within the countries. The N-CAP Process is a new tool that was developed for this purpose and has been piloted in the Eastern Mediterranean Region with potential use around the world. The N-CAP Process is a critical resource to advance global health security through multistakeholder collaboration to achieve more sustainable progress, a critical step forward in addressing today’s public health threats.

## Data availability statement

The original contributions presented in the study are included in the article/Supplementary material, further inquiries can be directed to the corresponding author.

## Author contributions

RS: Conceptualization, Methodology, Project administration, Writing – original draft, Writing – review & editing. RA: Writing – review & editing. MK: Conceptualization, Methodology, Project administration, Writing – original draft, Writing – review & editing. LK: Project administration, Writing – review & editing. SL-R: Methodology, Project administration, Writing – review & editing. PR: Conceptualization, Writing – review & editing. YK: Conceptualization, Methodology, Project administration, Writing – review & editing.
