# Supplementary material for: Addressing emerging public health threats: the Noncommunicable Disease Capacity Assessment and Planning (N-CAP) Process
Source: Front Public Health. 2024 Jun 5;12:1384957. doi: 10.3389/fpubh.2024.1384957 (PMC11188389; doi:10.3389/fpubh.2024.1384957)
Supplement: Supplementary file 1 [file Table_1.DOCX]

Supplementary Material

Table: Sections of the four Standard Operating Procedures (SOPs) modules of the Noncommunicable Disease Capacity Assessment and Planning (N-CAP) Process

| SOP Module | Roles and Responsibilities | | | | | Materials used or Additional Resources | Appendices |
| --- | --- | --- | --- | --- | --- | --- | --- |
|  | **National/regional public health organization** | **Ministry of Health** | **Facilitator(s)** | **Recorder(s)** | **Participating stakeholders** |  |  |
| Introduction and Preparation | - Promotes the N-CAP Process in their country or region - Introduces the MoH to the N-CAP Process and its expected outcomes - Identifies facilitators | **MoH Leadership^1^**:   - Work with the facilitators to determine if N-CAP Process is appropriate - Designate a POC   **MoH POC**^2^**:**   - Review the SOPs - Secure internal buy-in from MoH leadership | - Formally introduce the N-CAP Process with the interested countries - Ensure all materials are in the appropriate language - Lead introduction meeting and share SOPs with MoH POC and leadership | - N/A | N/A | - Summary of the N-CAP Process^3^ - N-CAP Process Background and Description Presentation^4^ | N/A |
| Stakeholder Mapping | N/A | **MoH POC**   - Has authority to review/revise Stakeholder Mapping Report | - Conduct mapping - Post-mapping, recommend participants for the SWOT Workshop - Write Stakeholder Mapping Report with input from MoH POC | - N/A | - N/A | - GHD\| EMPHNET Stakeholders’ Mapping and Analysis Toolkit^5^ | - Stakeholder Mapping Report Template |
| SWOT Workshop | - Observes Workshop to establish relationships for successful follow-up and to understand outcomes reached within the country | **MoH POC**   - Determines organizations to invite - Coordinates logistics/invitations with facilitators - Attends Workshop - Has authority to review SWOT Report | - Lead the Workshop - Post-SWOT, identify critical NCD area(s) (DGs) and recommend participants for the N-CAP Workshop with input from MoH POC - Write SWOT Report with input from MoH POC | - Document discussions during the SWOT Workshop - Write/type key information while the facilitator leads the discussion | - Select their representatives to participate - Share perspectives of their organization and discuss issues at a national level | - Facilitators use their own expertise to determine materials or tools to lead the Workshop - N-CAP Process Background and Description Presentation | - SWOT Report Template: For Participants - SWOT Report Template: For MoH |
| N-CAP Workshop | - Observes Workshop to establish relationships for successful follow-up and to understand outcomes reached within the country - Coordinates with MoH POC, MoH leadership, and facilitators, as agreed, to debrief after Workshop - Coordinates with MoH POC, MoH leadership, and N-CAP Workshop participants for follow-up in 6-12 months | **MoH POC**   - Works with facilitators to select the DGs - Determines organizations to invite - Coordinates logistics/invitations with facilitators - Attends Workshop - Has authority to review N-CAP Process Report   **MoH Leadership and MoH POC**   - Coordinates with public health organization and/or facilitators to debrief after Workshop - Coordinates with public health organization and N-CAP Workshop participants for follow-up in 6-12 months | - Lead the Workshop - Encourage discussions, summarize key results, and guide participants toward developing recommendations and/or plans for next steps - Facilitate Assessment Phase and Prioritization and Planning Phase for participants - Lead Transition with recorders - Conclude with Summary Wrap-Up for participants - Coordinate with recorders and external evaluator to debrief after Workshop - Write N-CAP Process Report - Coordinate with MoH POC, MoH leadership, and public health organization, as agreed, to debrief after Workshop | - Document discussions during the N-CAP Workshop - Write/type key information into the Assessment Form and Prioritization and Planning Form while the facilitator leads the discussion - Assist facilitators during Transition - Assist facilitators with writing N-CAP Process Report, as appropriate - Participate in debrief after Workshop with facilitators and external evaluator | - Select their representative to participate - Share perspectives of their organization and discuss issues at a national level - Participate in the Assessment Phase and Prioritization and Planning Phase with facilitator - Participate with MoH POC, MoH leadership, and public health organization for follow-up in 6-12 months | - N-CAP Process Background and Description Presentation - DGs - Assessment Form - Prioritization and Planning Form - External Evaluation Form (optional) | - Assessment Form - Prioritization and Planning Form - External Evaluation Form - N-CAP Process Report Template |
| Abbreviations: DGs Discussion Guides; MoH Ministry of Health; N-CAP Process Noncommunicable Diseases Capacity Assessment and Planning Process; POC Point of Contact; SOPs Standardized Operating Procedures.  Recommended to include those who lead NCD-related departments or teams and those involved with the strategic planning of the MoH across all departments.  ^2^ Should be within the NCD department or equivalent and have the authority to review the N-CAP SOPs and determine the country’s capacity to participate and effectively use the N-CAP Process to address NCDs.  ^3^ One-page, high-level document used to promote the N-CAP Process to interested countries and to summarize it to MoH leadership who may not be directly involved in all activities of the N-CAP Process.  ^4^ A PowerPoint presentation that describes the activities of the N-CAP Process, introduces the maturity model, explains the DGs, and provides an overview of the N-CAP Workshop. It is updated as each activity is completed to reflect the outcomes of that activity and later used to provide an overview of the Process in subsequent activities.  ^5^ Amiri M, Al Nsour M. (2021). GHD\|EMPHNET Stakeholders’ Mapping and Analysis Toolkit. Version 16.6. Available: <https://bit.ly/3BEBiga>.  ^6^ Used by recorders to note the important elements of the discussion, including participants’ input regarding the current and preferred level of progress, with their reasoning, as well as the issues to be addressed to reach the preferred level.  ^7^ Used by the recorders to categorize the issues identified in the Assessment Form and enable their prioritization and the planning of next steps with participants, including identification of who is responsible and timelines.  ^8^ Used by the external evaluator to document what is going well during the Workshop, and what needs improvement. | | | | | | | |
